# Virtual articulator for the analysis of dental occlusion: An update

**DOI:** 10.4317/medoral.17147

**Published:** 2011-12-06

**Authors:** Laura Maestre-Ferrín, Javier Romero-Millán, David Peñarrocha-Oltra, María Peñarrocha-Diago

**Affiliations:** 1 DDS. Master in Oral Surgery and Implantology. Faculty of Medicine and Dentistry. University of Valencia; 2 DDS. Resident of the Master in Oral Surgery and Implantology. Faculty of Medicine and Dentistry. University of Valencia; 3 Associated Professor of Oral Surgery. Faculty of Medicine and Dentistry. University of Valencia. Valencia (Spain)

## Abstract

The future of dental practice is closely linked to the utilization of computer-based technology, specifically virtual reality, which allows the dental surgeon to simulate true life situations in patients. The virtual articulator has been designed for the exhaustive analysis of static and dynamic occlusion, with the purpose of substituting mechanical articulators and avoiding their errors. These tools will help both odontologists and dental prosthetists to provide the best individualized treatment for each patient. The present review analyzes the studies published in the literature on the design, functioning and applications of virtual articulators. A Medline-PubMed search was made of dental journals, with the identification of 137 articles, of which 16 were finally selected. The virtual articulator can simulate the specific masticatory movement of the patient. During mandibular animation, the program calculates the sites where the opposing teeth come into contact. The studies made to assess the reliability of the virtual articulator show good correspondence in visualization of the number and position of the dynamic contacts. The virtual articulator is a precise tool for the full analysis of occlusion in a real patient.

** Key words:** Virtual articulator, dental occlusion, dental articulator.

## Introduction

The virtual articulator has been designed for the exhaustive analysis of static and dynamic occlusion. This tool incorporates virtual reality applications to the world of dental practice with the purpose of replacing mechanical articulators and thereby avoiding the errors and limitations of the latter (1).

In daily practice, mechanical articulators are used to diagnose and simulate the functional effects of malocclusions and morphological alterations upon dental occlusion. However, this mechanical scenario, so very different from the real life biological setting, poses a series of problems. In effect, the movements reproduced by the mechanical articulator follow the margins of the structures that conform the mechanical joint, which remain invariable over time, and which cannot simulate masticatory movements that are dependent upon the muscle patterns and resilience of the soft tissues and joint disc. Moreover, tooth mobility cannot be simulated by plaster models; as a result, the latter are unable to reproduce the real life dynamic conditions of occlusion (2,3). There are also other problems derived from the procedures and materials used for assembling the models in the articulator: precision in orienting the model, expansion and contraction of the plaster, deformation of the bite-recording material, the stability of the articulator, etc. (3).

Because of these factors, dynamic reproduction of the excursive movements appears to be scantly reliable. In this sense, Tamaki et al. (4) reported that only 82% of the teeth reproduce the protrusive contacts, and that 90% reproduce the laterality contacts in the mechanical articulator adjusted by computed axiography. In addition, the mechanical articulator generates new contacts (5). 

The virtual articulator offers the possibility of significantly reducing the limitations of mechanical articulators (6), due to a series of advantages: full analysis can be made of static and dynamic occlusion, of the inter-maxillary relationships, and of the joint conditions, thanks to dynamic visualization in three dimensions (3D) of the mandible, the maxilla or both, and to the possibility of selecting section planes allowing detailed observation of regions of interest such as for example the temporomandibular joint (3). Combined with CAD/CAM technology, this tool offers great potential in planning dental implants, since it affords greater precision and a lesser duration of treatment (1).

The present review analyzes the data found in the literature on the design, functioning and applications of virtual articulators.

## Search Strategy

An automated PubMed search was made, using the following key words: “virtual articulator” and “virtual occlusion”. The search identified 137 articles, of which 16 were finally selected. We only included those articles that referred to functioning of the virtual articulator in analyzing dental occlusion in a real patient real, and which compared the virtual articulator versus its mechanical counterpart with a view to assessing the validity of the former.

## Patient registries for the Virtual Articulator: Input data

The programming and adjustment methods of the virtual articulator were described by Kordass and Gärtner in 1999. First a digital image is obtained of the surfaces of each tooth, of the global dental arches, and of the bite registries. To this effect a three-dimensional laser scanner is used, such as for example the Laser Scan 3D (Willytec, Munich, Germany). This scanner projects a vertical laser beam onto the surface of the object. A digital camera equipped with a charge coupled device (CCD) registers the beam reflected from the object and transmits the digital signals to an electronic processing system. The processed image data are stored as digital matrix brightness values, ready for use by the scanner software and for on-screen visualization and computerized manipulation (7).

Many systems are available for recording the mandibular movement pattern. It has been demonstrated that mandibular movements cannot be simplified in terms of pure rotation around the condylar axis (8). Gärtner and Kordass (7), and Bisler et al. (1), used an ultrasound system to analyze mandibular movement: the Jaw Motion Analyser® (JMA) (Comp Zebris, Isny, Germany). This device consists of an ultrasound source bound to the labial surface of the mandible by means of a customized accessory, and a sensor system housed in another accessory mounted round the head of the patient in the form of a facial arc. The operator defines a plane by means of the coordinates of the two condylar points and an infraorbital point. The system stores the movements of at least three points relative to this plane. Specifically, these three points comprise the two condylar points and the center of the base of the accessory joined to the mandible. In this way it is possible to detect rotation and exact position with all the degrees of freedom (9). 

There are also other systems for the detection of mandibular movements based on other technologies, such as optoelectronic devices that use CCD cameras to register the emissions of light-emitting diodes (LEDs) positioned over the head of the patient and generate an image from these signals (10). Fang and Kuo (10), using this system, presented a new model for assessing mandibular dynamics. They designed an individualized device for each patient, fixed in the same position in both the plaster models and in the oral cavity. First the models were scanned, and then the patients were instructed to perform the three types of mandibular movement (aperture / closure, protrusion / retrusion, and lateralization) at least 20 times. The data obtained were in turn processed by mathematical models to reconstruct the individual dynamics of each patient for visualization and computer-based analysis. 

## Functioning of the Virtual Articulator

The basic system of the virtual articulator generates an animation of the movements of the mandible based on the input data, and calculates the points of occlusion, which in turn are shown on-screen by means of some type of code (3).

Ideally, the virtual articulator is equipped with a device for registering the specific mandibular movements of a given patient (such as the JMA), and can integrate the movements recorded in the animation. 

If no device is available for registering the mandibular movements, specific movements must be defined by means of parameters, in a way similar to the practice used with mechanical articulators. Some parameters of interest in these cases would be the following: protrusion (radius of the condylar guide, maximum distance of condylar protrusion), retrusion (radius of the condylar guide, maximum distance of retrusion), laterotrusion (maximum protrusion, Bennett angle, radius of the right and left condylar guide, right and left horizontal condylar slope, phase angle, lateral displacement), and aperture / closure slope (maximum angle of aperture). After defining the movement parameters, collision detection is required in order to identify the movement restrictions. In these cases, it may be of interest to leave a distance corresponding to the thickness of the occlusion paper used in the mechanical articulators, for calculating the points of occlusion on the basis of this distance (3).

As an example, the software of the Dent-CAM® virtual articulator (Comp. KaVo, DLeutkirch) uses three main windows that show the same movement pattern, distinguishing a series of aspects: a) interpretation window: this shows both maxilla in dynamic occlusion and allows us to obtain unusual points of view, e.g., observation from an occlusal surface of closing of the opposing tooth during mastication; b) occlusion window: this shows the points of contact that appear on the occlusal surfaces of the upper and lower teeth as a function of time; and c) section window: this offers different frontal sections along the dental arch. This tool can be used to analyze the degree of intercuspidation, as well as the height and functional angles of the cuspids. 

The latest software versions incorporate an orthodontic module allowing the creation of a virtual setup. The program has also been equipped with the representation of the condylar trajectories in the sagittal and horizontal planes. This tool allows us to observe the interrelationship between the incisal guide and the condylar guide, and the effects of joint mobility upon occlusion (7).

One of the most recent new developments in the virtual articulators is the 3D virtual articulation system (Zebris company, D-Isny). This system requires the following: a) an input unit in the form of a 3D scanner; b) the software for prosthesis modeling and collision detection, based on a virtual articulator; and c) the output module (a rapid prototyping system). With this system, and in addition to mandibular movement, we can analyze masticatory movement – including force at the points of contact and the frequency of contacts in relation to time (11).

## Validation

On comparing the DentCAM® virtual articulator with a mechanical articulator (Comp. KaVo, DLeutkirch), approximately the same number of contacts were obtained in the lateralization movements with both articulators in 8 patients (mechanical articulator: 90; virtual articulator: 92) (3). In order to assess reliability, three operators measured the mandibular movements twice in 8 subjects. Good correspondence was found in visualization of the number and position of the dynamic contacts (3).

In this same line, Pröschel et al. (12) carried out a study of 57 asymptomatic patients in order to determine the occlusal errors appearing in the mechanical articulators. To this effect comparisons were made with the virtual articulator, yielding an error in the second molar of 200 µm in 16% of the patients and of 300 µm in 6% of the subjects – this implying a low risk of error, though the acceptable limits in clinical practice could be exceeded.

Likewise, other studies have compared the maximum number of contacts between the conventional method and the virtual articulator – the occlusal contacts calculated from the virtual models being shown to precisely reproduce the contacts obtained with the mechanical articulator (13,14).

The virtual articulator has also been compared with the mechanical articulator in orthognathic surgery, to establish ideal maxillary position and for preparing surgical splints. Song and Baek (15) carried out a study in 25 patients previously subjected to orthodontic treatment and who were programmed for Le Fort 1 fracture in the maxilla and a sagittal osteotomy in the mandible. The authors compared the precision of the surgical model and of the splints, concluding that the virtual method is more precise than the conventional approach – though there were no significant differences between them. In this same line, Ghanai et al. (16), in 6 patients programmed for repositioning of one or both maxillas, compared the deviation between the two methods using MicroScribe G2X (Immersion Corporation, San Jose, CA, USA). The authors concluded that the virtual articulator can precisely reproduce conventional planning and help inexperienced surgeons to obtain good results.

## Conclusion

The virtual articulator is a precise tool for the full analysis of occlusion in a real patient, and can help the dental professional in establishing a diagnosis and in planning the best treatment option.
